# Modified Circular Hemorrhoidectomy for Stage IV Hemorrhoids With Terminal Prolapse: A Case Series

**DOI:** 10.7759/cureus.81394

**Published:** 2025-03-28

**Authors:** Arturs Niedritis, Jans Ameriks, Sergejs Lebedjkovs

**Affiliations:** 1 Department of General Surgery, Riga Stradins University, Riga, LVA; 2 Department of General Surgery, AIWA Clinic, Riga, LVA

**Keywords:** anal prolapse, hemorrhoidal disease, modified circular hemorrhoidectomy, mucosal bridges, proctology, whitehead hemorrhoidectomy

## Abstract

Stage IV hemorrhoids with terminal prolapse present a significant surgical challenge, often requiring innovative approaches to achieve optimal outcomes. This case series highlights the efficacy of modified circular hemorrhoidectomy, a refined technique incorporating mucosal bridge preservation, in three patients with severe circumferential hemorrhoidal disease. The procedure, adapted from the traditional Whitehead hemorrhoidectomy, addresses both internal and external components while minimizing postoperative complications such as anal stenosis and mucosal ectropion. All three patients experienced complete resolution of complaints, with no recurrence or major complications during follow-up. The modified technique not only demonstrated technical feasibility but also improved functional outcomes and patient satisfaction. This series underscores the potential of modified circular hemorrhoidectomy as a valuable surgical option for stage IV hemorrhoids with terminal prolapse, offering a balance between radical excision and preservation of anatomical integrity. Further studies are warranted to validate these findings and establish their role in modern hemorrhoidal disease management.

## Introduction

Hemorrhoidal disease, a prevalent condition affecting millions worldwide, has seen significant evolution in its surgical management over the past century. Among the various techniques developed, the Whitehead hemorrhoidectomy, introduced in 1882, emerged as a prominent method for treating severe hemorrhoidal disease, particularly in cases of circumferential lesions with terminal prolapse. However, its popularity decreased in the mid-20th century due to reports of high postoperative complication rates, including anal stenosis and mucosal ectropion [1,2]. As a result, the procedure was largely abandoned in favor of alternative techniques, such as stapled hemorrhoidopexy and excisional hemorrhoidectomy, which were perceived as safer and more effective.

Despite its decline, recent studies have rekindled interest in the Whitehead hemorrhoidectomy, particularly in its modified form. The modified Whitehead hemorrhoidectomy incorporates the preservation of mucosal bridges, which refers to the technique in which intact mucosal bridges between excised hemorrhoidal segments are preserved to prevent circumferential loss of mucosa, a technical refinement that has been shown to significantly reduce the risk of postoperative complications, such as anal stenosis and skin tag formation [3]. This adaptation has demonstrated particular efficacy in treating Grade IV hemorrhoidal disease with circumferential terminal prolapse, characterized by permanently prolapsed hemorrhoidal tissue that is irreducible, a condition that often proves challenging to manage with other surgical techniques [4].

Traditional methods, such as stapled hemorrhoidopexy and excisional hemorrhoidectomy, while effective for many patients, often fall short in addressing the complexities of circumferential lesions and terminal prolapse. Stapled hemorrhoidopexy, for instance, may not adequately resolve external components of hemorrhoidal disease, while excisional techniques can lead to significant tissue loss and functional impairment [5,6]. In contrast, the modified Whitehead hemorrhoidectomy offers a more comprehensive solution by addressing both internal and external components while preserving mucosal integrity, thereby reducing the risk of complications and improving patient outcomes [7,8].

This resurgence in the use of the modified Whitehead hemorrhoidectomy underscores the importance of revisiting historical techniques with modern refinements. By incorporating mucosal bridge preservation, the procedure not only mitigates the shortcomings of the original technique but also provides a viable option for patients with severe, circumferential hemorrhoidal disease that may not be fully addressed by other methods. The following case series explores the application of this modified approach in patients with Grade IV hemorrhoidal disease, highlighting its potential as a valuable tool in the surgical management of this challenging condition.

## Case presentation

Patient 1

The patient was a 45-year-old female who presented with a long-standing history of symptomatic hemorrhoidal disease, progressively worsening over the past five years. She reported severe discomfort, itching, bleeding, pain, and difficulty with defecation, alongside a persistent sensation of incomplete evacuation. The patient noted the necessity to reposition the hemorrhoids back into the anal canal after defecation. The symptoms significantly impacted her quality of life, limiting daily activities and leading to social withdrawal; as an example, the patient was afraid of defecating in public restrooms due to possible bleeding and stained clothes after wiping.

On clinical examination, a circumferential prolapse of internal and external hemorrhoids consistent with Grade IV hemorrhoidal disease was observed. The prolapsed tissue was edematous, with evidence of chronic irritation and superficial ulceration. Digital rectal examination revealed no additional masses or abnormalities beyond the prolapse, and anal sphincter tone was within normal limits.

Despite prior conservative management, including dietary modifications, topical agents, and band ligation therapy, the patient's symptoms had not resolved; therapy was started with a delay due to perceived stigmata of hemorrhoidal disease. Given the advanced stage of the disease and failure of nonsurgical interventions, surgical management was indicated.

The patient had undergone preoperative colon preparation using a laxative suppository, "Microlax," three times the previous day. All operations were performed by a board-certified senior general surgeon with 40 years of experience in colorectal surgery.

A modified circular hemorrhoidectomy was planned, incorporating adjustments like preservation of healthy mucosal bridges to the standard technique to address the circumferential prolapse while preserving anal function and minimizing postoperative complications. This method was chosen due to the inability of more common techniques like the Longo procedure, Milligan-Morgan, or Ferguson hemorrhoidectomy to completely address the issue, which would lead to poor patient satisfaction, relapse, and incomplete resolution of symptoms.

**Figure 1:**
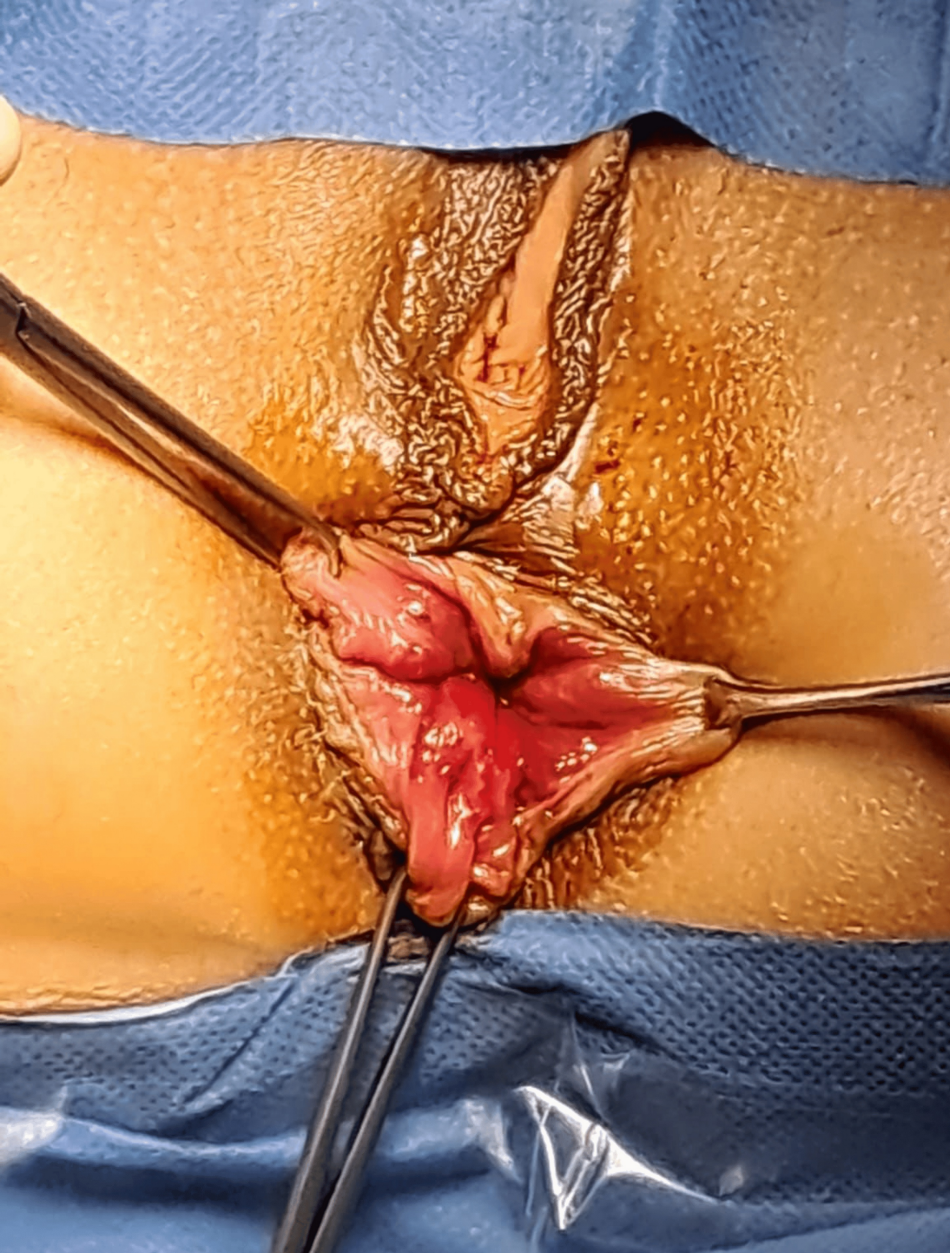
Beginning of the operation It is possible to visualize the circumferential nature of the hemorrhoidal plexuses, with protrusion of internal plexuses, as well as external plexuses and skin tags.

**Figure 2:**
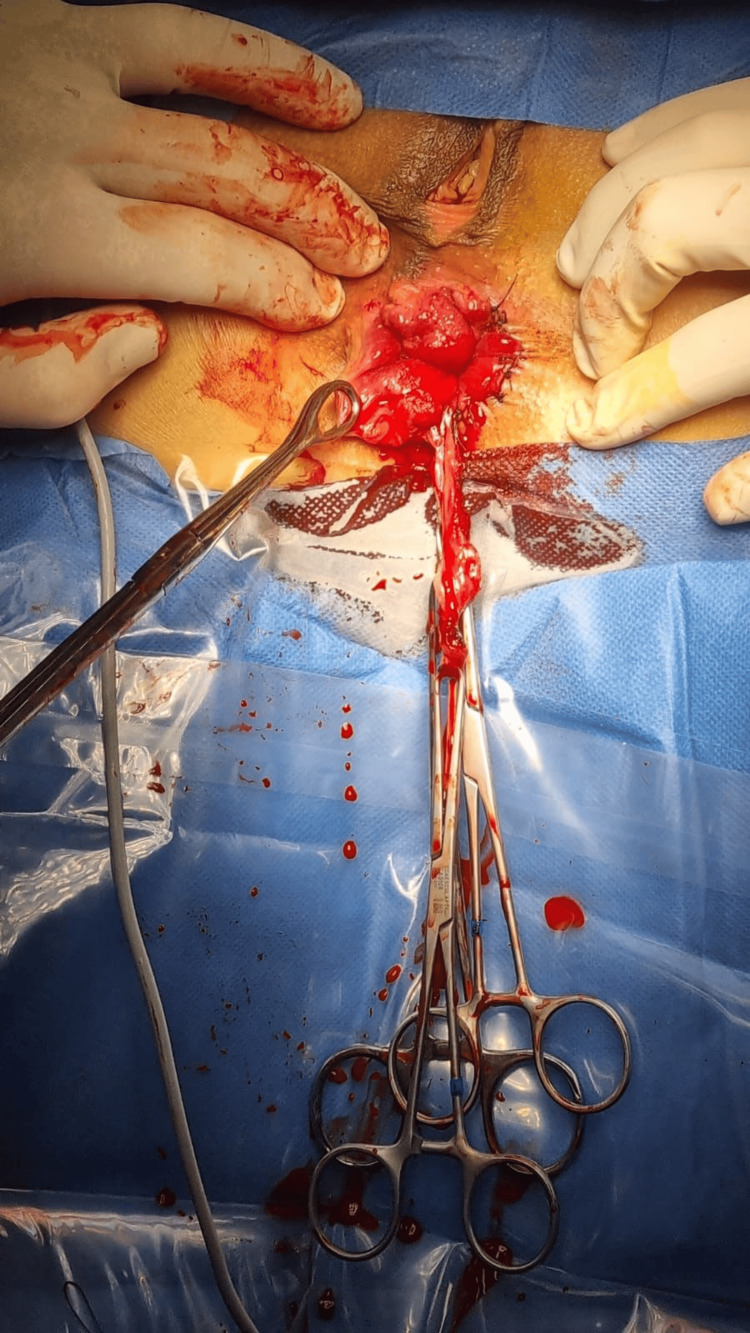
Comparison of operated and unoperated sides

**Figure 3:**
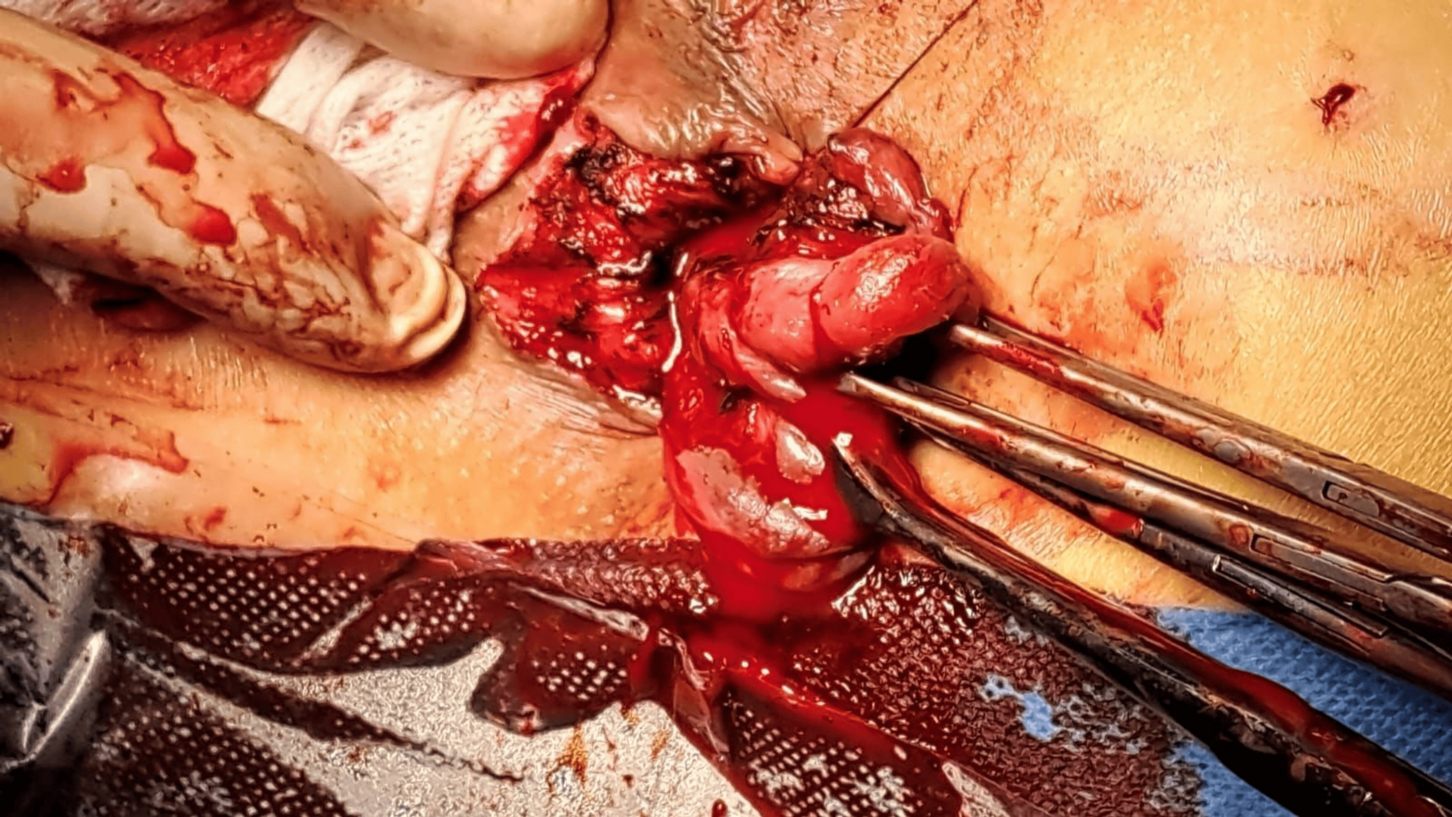
Identification of anal sphincter The most important part of this technique is to visualize the anal sphincter to avoid damage; otherwise, the chance of serious complications, like anal incontinence, is likely.

**Figure 4:**
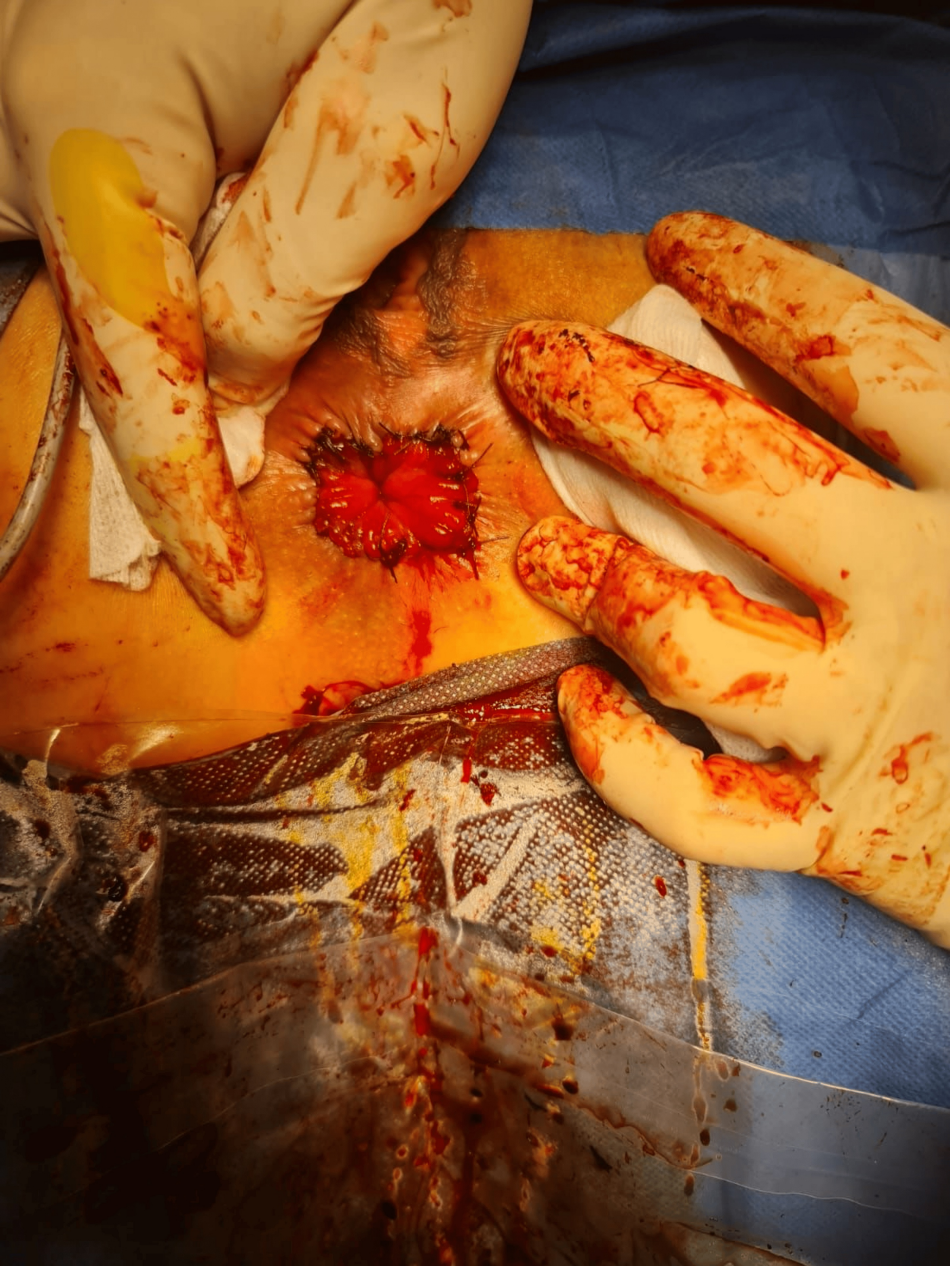
Result immediately after operation Complete excision of hemorrhoidal tissues and no prolapse is observed.

Anal dilation was performed using a 34 mm dilator for 10 minutes before incision to reduce hypertonicity of the internal anal sphincter, to prevent stenosis, and to reduce postoperative pain. The procedure lasted for 53 minutes under general anesthesia. Depth of hemorrhoids was assessed and healthy mucosa was identified, then a semi-circle incision was performed around the anal canal first on the right side (Figures 1-2). Next, blunt dissection and careful hemostasis of bleeding tissues were performed to reliably visualize the internal and external anal sphincter and prevent possible injury; from there, mucosa was dissected until all hemorrhoidal plexuses were excised (Figure 3). The wounds were sutured radially by a simple interrupted suture to prevent dehiscence and minimize tissue ischemia, and the same technique was performed on the opposite side. It was possible to discern between hemorrhoidal plexuses and several unaffected mucosal regions where mucosal bridges were left. The closure of the wounds was performed using a multifilament absorbable 3-0 suture. There were no hemorrhoidal plexuses observed on postoperative evaluation (Figure 4). The surgical wound was covered by a hemostatic sponge for the first 24 hours. After surgery, the patient was treated with intravenous analgesia for 12 hours and broad-spectrum antibiotics for the first 24 hours, which were switched to the oral regimen the day after surgery. The patient was discharged on postoperative day 1. At the postoperative outpatient follow-up in the first, second, and fourth weeks, the patient reported no bothering symptoms like pain, bleeding, anal incontinence, or recurrence of other previous complaints. In addition, anal stenosis and wound dehiscence were not identified on physical examination. All patients were scheduled for follow-up visits after three, six, and 12 months.

**Figure 5:**
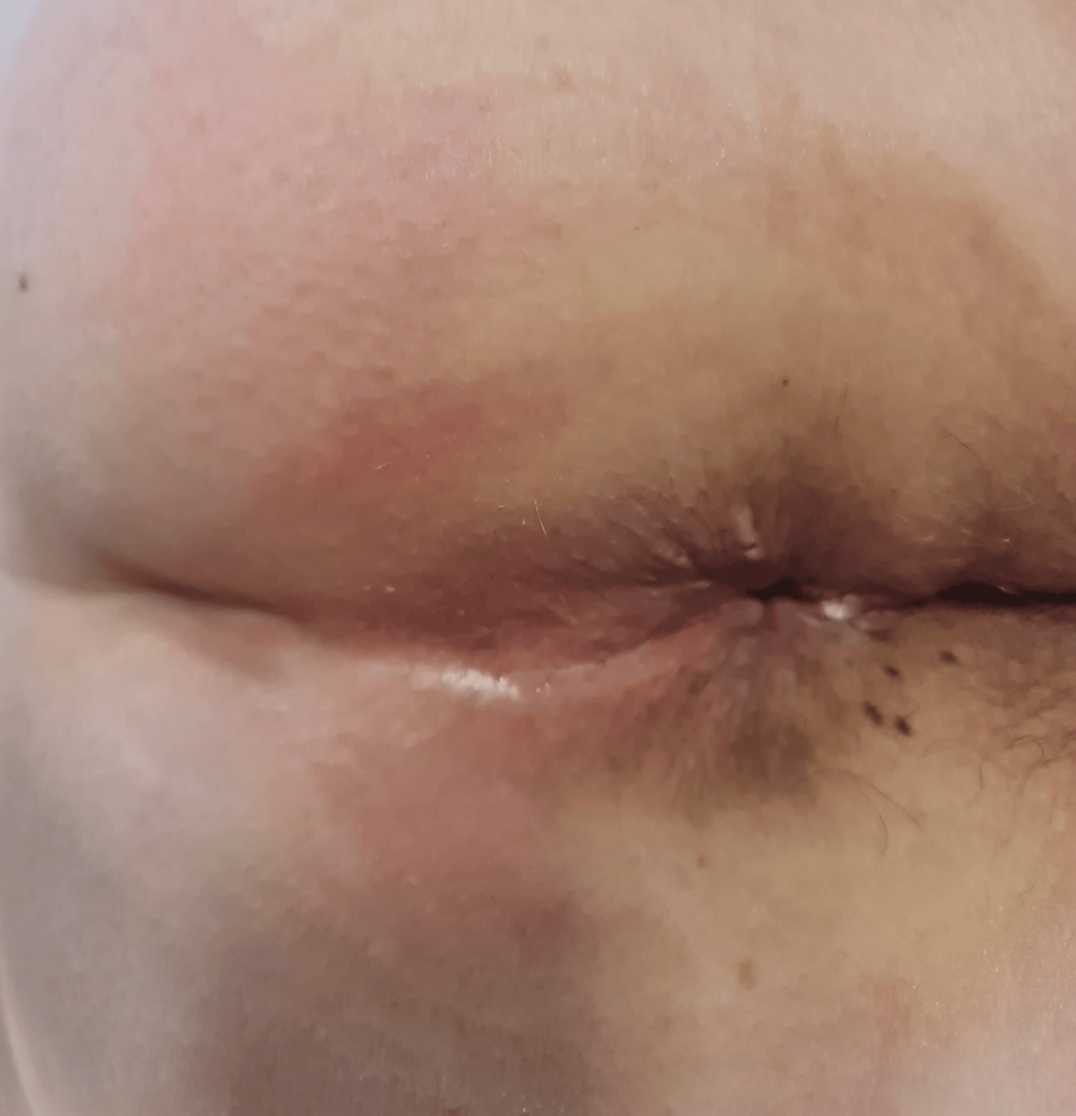
Patient follow-up after three months Profound cosmetic improvement is observed; also, no recurrence of hemorrhoids is observed, and no mucosal ectropion. Digital rectal examination shows no anal stenosis.

Postoperatively, the patient was managed with a regimen emphasizing pain control, wound care, and prevention of constipation. She experienced significant symptomatic relief, with resolution of bleeding and prolapse, and reported substantial improvement in quality of life during follow-up visits. Anal function was preserved, with no evidence of stenosis or incontinence at the three-month follow-up (Figure 5).

This case underscores the potential of a tailored surgical approach, such as the modified circular hemorrhoidectomy, in managing complex cases of advanced hemorrhoidal disease, especially with circumferential lesions and terminal prolapse.

Patient 2

The patient was a 48-year-old male who presented with a five-year history of symptomatic hemorrhoidal disease. He reported severe anal pain, bleeding, and itching, coupled with persistent prolapse after defecation, which he manually reduced. These symptoms escalated over the past year, with difficulty in maintaining hygiene. The impact on his personal life was profound, including social withdrawal and avoidance of outdoor activities.

**Figure 6:**
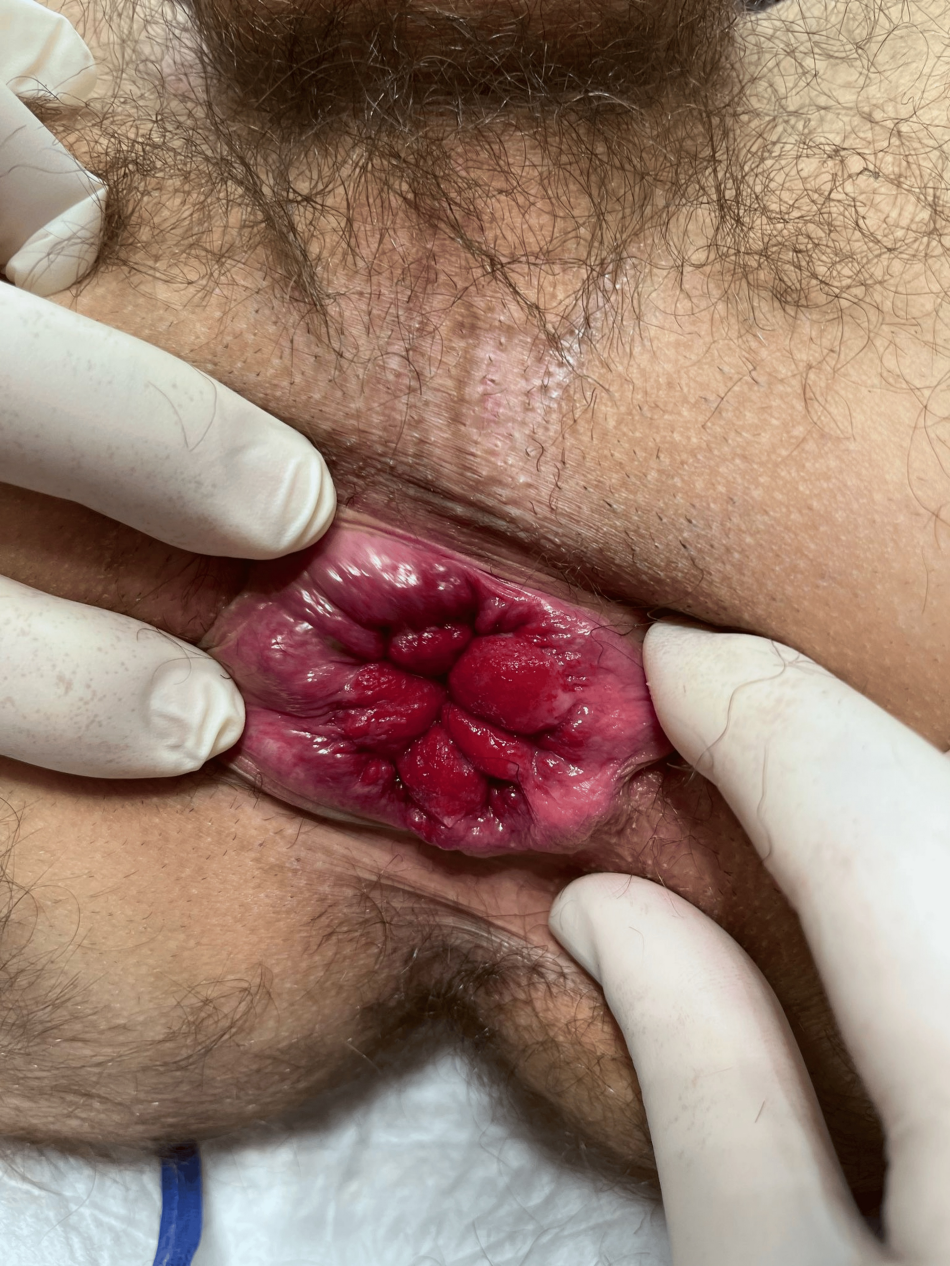
Before operation Visualization of prolapsing circumferential hemorrhoidal tissue.

Clinical examination revealed circumferential Grade IV hemorrhoidal prolapse with edematous and ulcerated tissue (Figure 6). Digital rectal examination showed no additional abnormalities, and anal sphincter tone was normal. Despite attempts at conservative management, including fiber supplementation and topical agents, his symptoms persisted. Surgical intervention was recommended due to the failure of these measures and the severity of his condition. It is important to note the perceived stigma of hemorrhoidal disease, especially in the male population, which may lead to late diagnosis and treatment.

**Figure 7:**
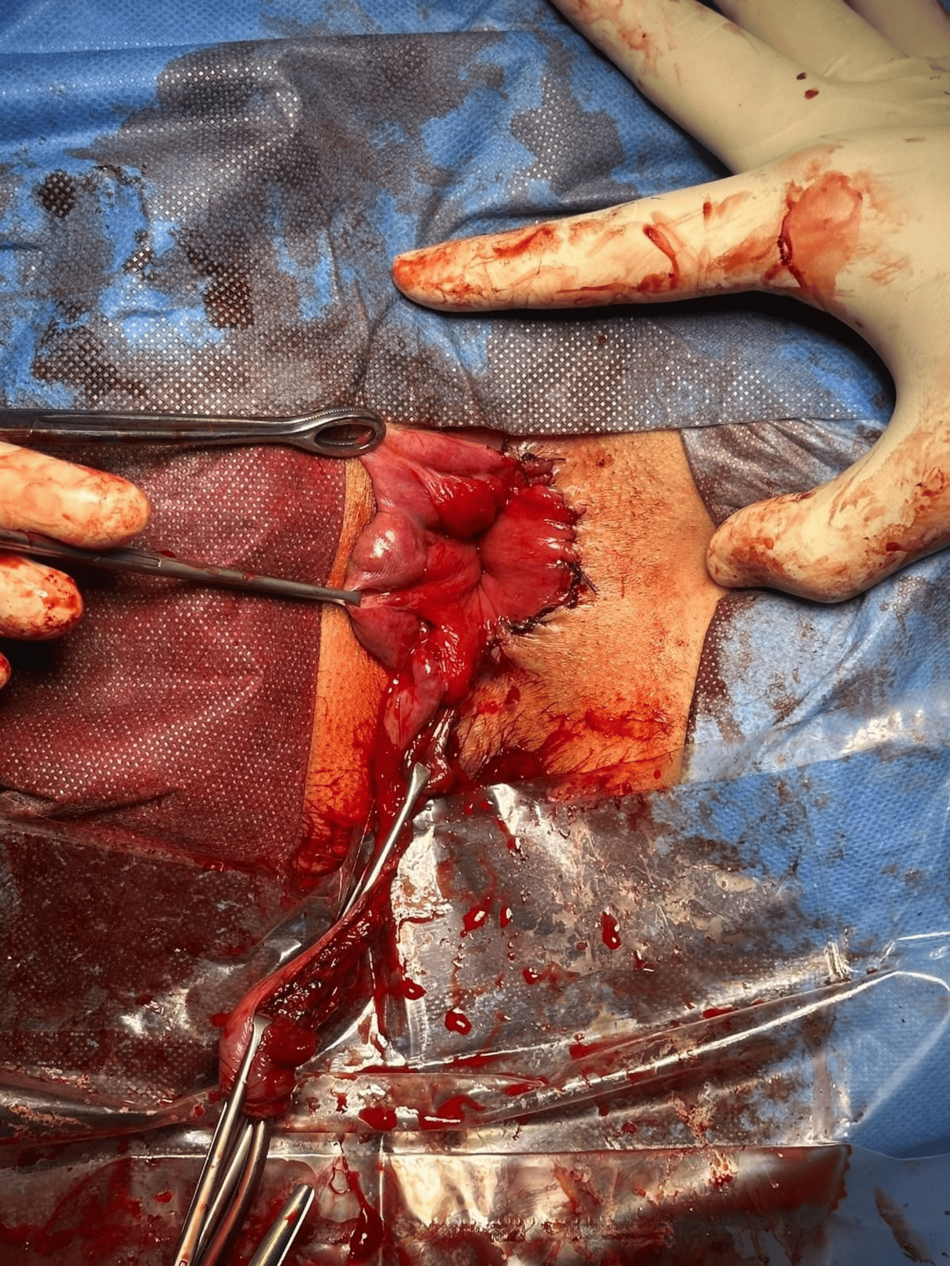
Comparison between operated and unoperated sides On the right side, it is possible to visualize healthy mucosa sutured to skin, with complete excision of both external and internal hemorrhoidal plexuses.

**Figure 8:**
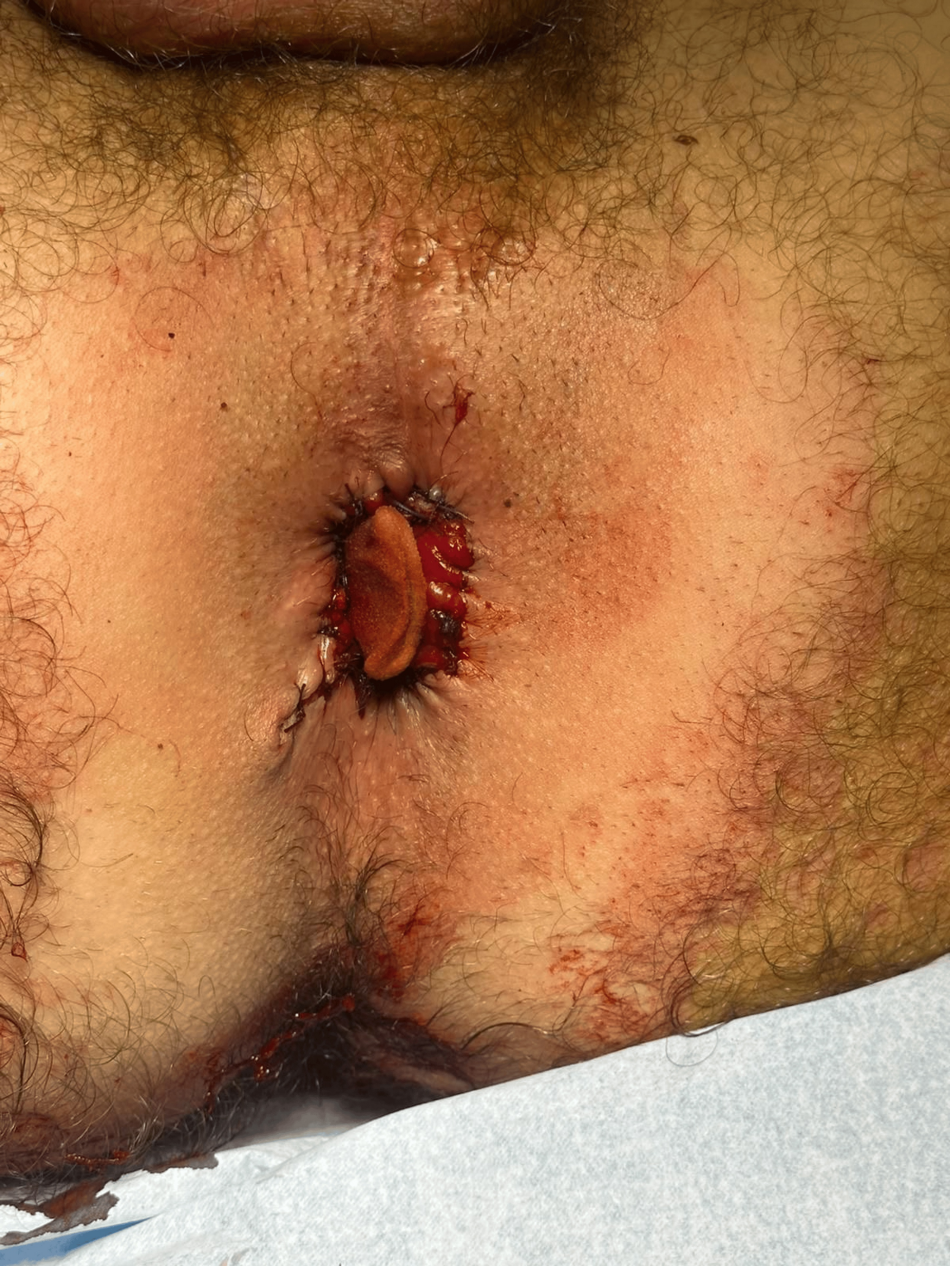
Result immediately after operation Completely excised hemorrhoidal tissue, with complete resolution of prolapse.

**Figure 9:**
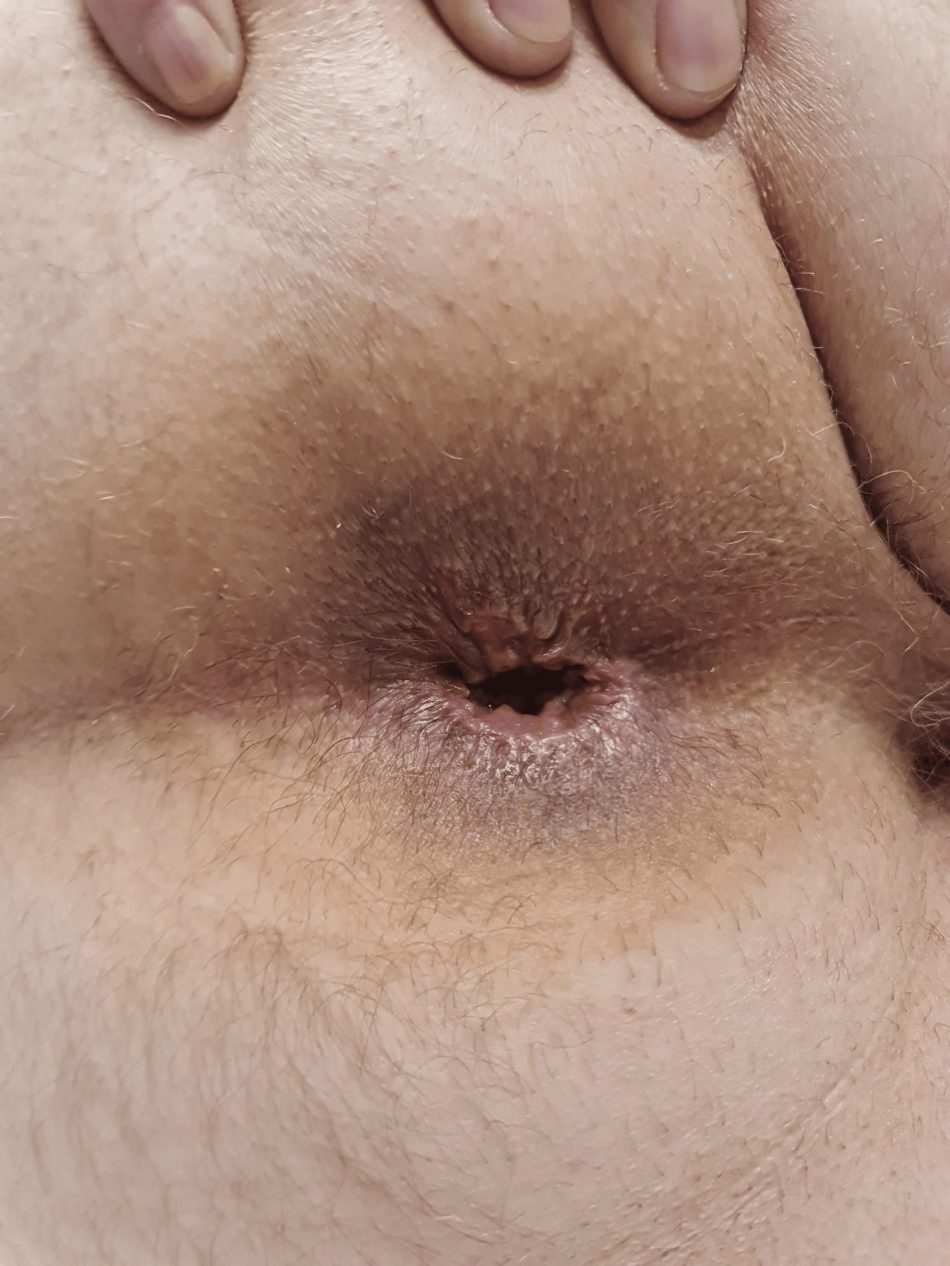
Follow up after three months On follow-up, no recurrence is seen, also no mucosal ectropion is visualized, and on digital rectal examination, no stenosis is observed. The patients are also happy with the cosmetic result.

The patient underwent a modified circular hemorrhoidectomy under general anesthesia. The procedure included the preservation of mucosal bridges and anal dilation to address hypertonicity of the internal sphincter. The procedure lasted 58 minutes under general anesthesia (Figures 7-8). Postoperative care included pain management, broad-spectrum antibiotics, and a high-fiber diet. The patient reported complete symptom resolution at follow-up visits over three months, with no incontinence, stenosis, or recurrence of prolapse (Figure 9).

Patient 3

The patient was a 55-year-old man with a seven-year history of progressive hemorrhoidal symptoms. His primary complaints included constant prolapse with inability to reposition, severe pain during defecation, and frequent bleeding episodes that required medical attention. The condition had become debilitating, preventing him from working and participating in routine activities due to constant discomfort.

**Figure 10:**
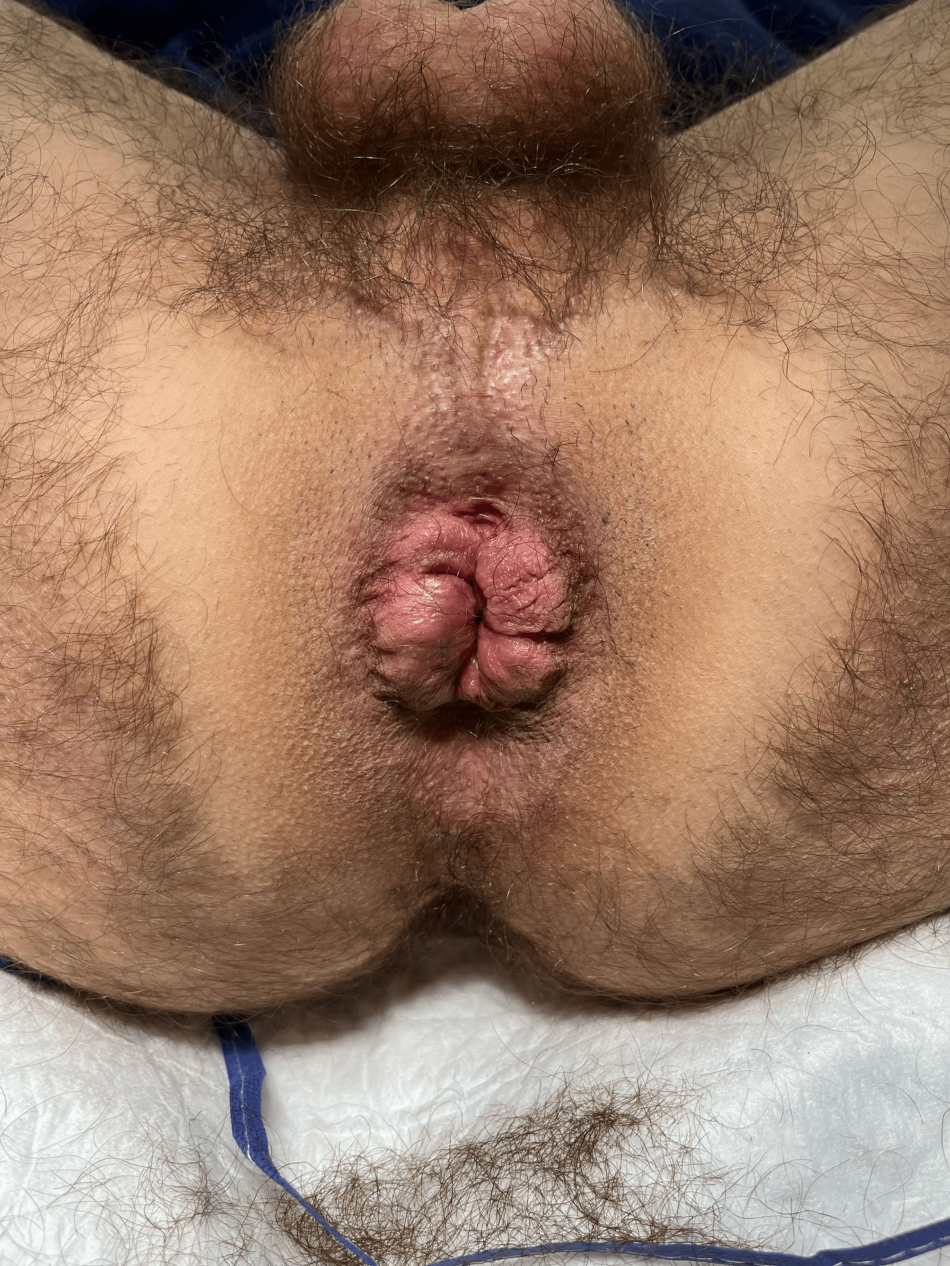
Before operation Protruding circular hemorrhoidal tissues.

**Figure 11:**
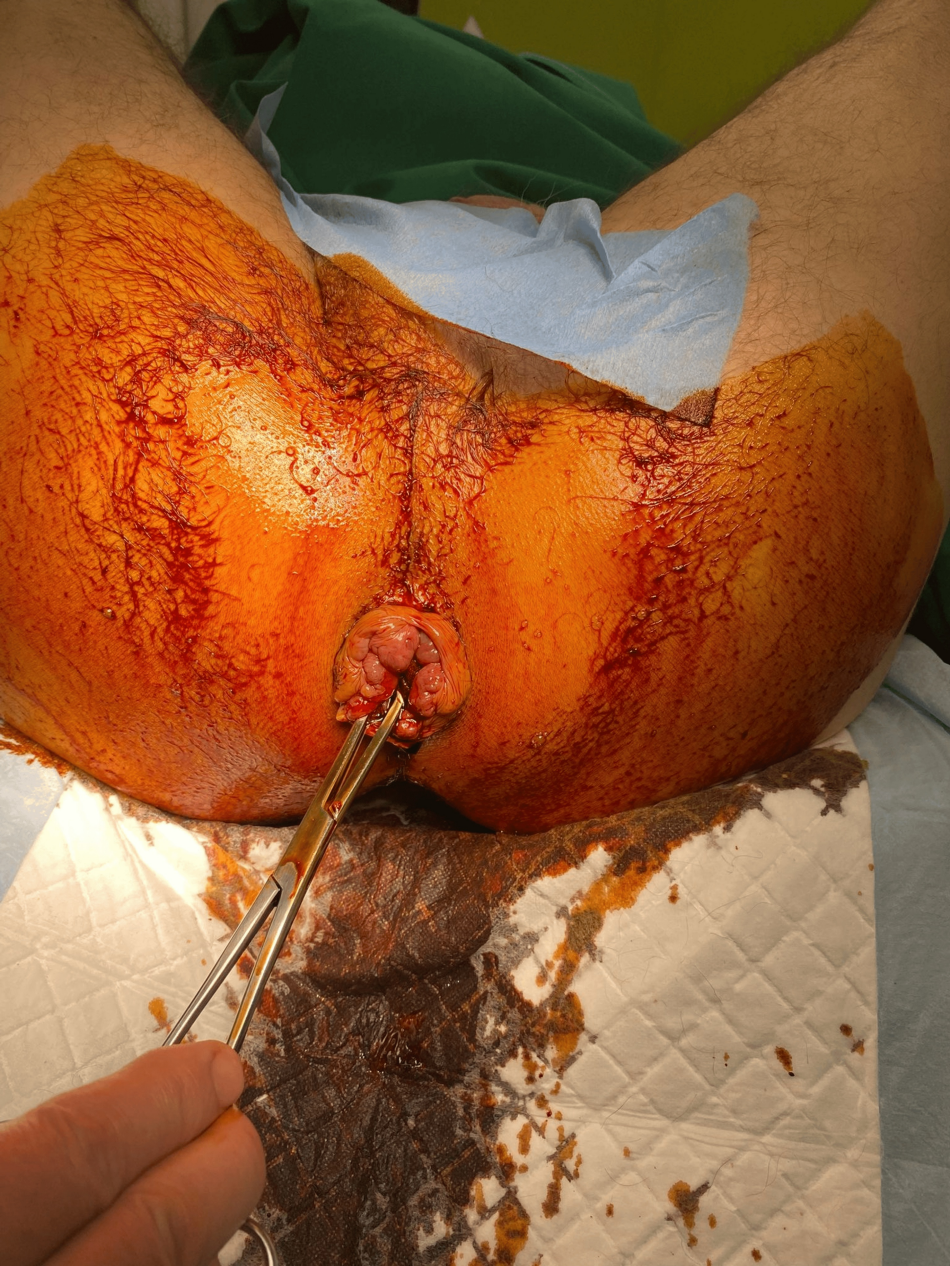
Prolapsing internal hemorrhoids It is also possible to visualize here the protrusion of internal hemorrhoidal plexuses.

Physical examination confirmed Grade IV hemorrhoidal disease with circumferential prolapse, significant edema, and chronic irritation. Rectal tone and sensation were within normal limits, and there was no evidence of other anorectal pathology. The patient had previously undergone failed band ligation therapy and reported limited adherence to conservative measures due to embarrassment (Figures 10-11).

He underwent a modified circular hemorrhoidectomy, during which mucosal bridges were preserved. The procedure lasted 65 minutes under general anesthesia. Anal dilation was performed to minimize postoperative pain and stenosis. The surgical wounds were sutured using absorbable 3-0 sutures, and a hemostatic sponge was applied. Postoperatively, the patient experienced rapid symptom relief and improved quality of life. At three months, he reported no recurrence, incontinence, or other complications.

## Discussion

Stage IV hemorrhoidal disease with terminal prolapse represents one of the most challenging scenarios in proctological surgery, requiring a delicate balance between radical excision of pathological tissue and preservation of anatomical and functional integrity. The modified circular hemorrhoidectomy, as described in this case series, offers a refined approach to addressing severe circumferential hemorrhoidal disease. By incorporating mucosal bridge preservation, this technique decreases the risk of postoperative complications such as anal stenosis and mucosal ectropion, which are commonly associated with Whitehead hemorrhoidectomy [2,4]. The favorable outcomes observed in all three patients - complete resolution of prolapse, absence of recurrence, and no major complications - underscore the efficacy and potential safety of this modified approach. In our center experience no cases of anal stenosis, anal incontinence, wound dehiscence or mucosal ectropion have occurred. The recurrence of interest in this technique is understandable, especially when it is evident that other traditional methods provide suboptimal results in cases of Grade IV hemorrhoidal disease. It is also important to note that many new surgeons are not taught, to our best knowledge, this technique due to historical association with severe complications and subsequent prioritization of other techniques, yet these cases illustrate the potential necessity to be able to perform these operations, if the best result is desired.

One of the key advantages of this technique is its ability to address both the internal and external components of hemorrhoidal disease while maintaining the structural and functional integrity of the anal canal. A key technical and surgical point to reduce the risk of surgical complications is the preservation of mucosal bridges, which ensures adequate mucosal coverage, reducing the risk of stenosis and promoting better wound healing [3]. It is of utmost importance to identify internal and external anal sphincters during the operation to prevent trauma to these muscles and to avoid subsequent complications. This anatomical precision is critical in achieving optimal functional outcomes and patient satisfaction.

The success of this technique also relies on effective teamwork, particularly with the anesthesiology team. Achieving optimal muscle relaxation throughout the procedure is paramount to prevent mucosal tearing during dissection, excision, and suturing. Inadequate relaxation can lead to increased tension in the anal canal, making the tissue more susceptible to injury. Close collaboration with the anesthesiology team ensures that the patient remains adequately relaxed, facilitating a smoother and safer surgical process. Relaxation is especially important during the suturing of anal mucosa to skin. Otherwise, mucosal tears will happen, and too radical excision and suturing will be needed, leading to increased risks of mucosal ectropion.

## Conclusions

Stage IV hemorrhoidal disease with terminal prolapse represents a complex surgical condition that demands innovative techniques to achieve both anatomical correction and functional preservation. The modified circular hemorrhoidectomy, as highlighted in this case series, offers a refined approach to managing severe circumferential hemorrhoidal disease. The complete resolution of prolapse, absence of recurrence, and lack of major complications in all three patients during short-term follow-up is an important stepping stone to consider this operation as a safe alternative to classical methods. Balance between radical excision and anatomical conservation is critical to achieving optimal outcomes in patients with advanced hemorrhoidal disease.

The success of the modified circular hemorrhoidectomy in the treatment of advanced hemorrhoidal disease also highlights the importance of interdisciplinary teamwork, particularly with the anesthesiology team. Optimal muscle relaxation throughout the procedure is essential to prevent mucosal tearing and to facilitate a smooth dissection process. Inadequate relaxation can increase tension in the anal canal, complicating the identification of anatomical structures and increasing the risk of tissue injury. The favorable functional outcomes and high patient satisfaction observed in this series of findings suggest potential benefits, but further studies are needed. However, while these results are promising, further studies with larger cohorts and longer follow-up periods are needed to establish the modified circular hemorrhoidectomy as a standard option in the surgical management of stage IV hemorrhoidal disease with terminal prolapse.
